# Rice ( *Oryza*) hemoglobins

**DOI:** 10.12688/f1000research.5530.2

**Published:** 2014-12-11

**Authors:** Raúl Arredondo-Peter, Jose F. Moran, Gautam Sarath

**Affiliations:** 1 Laboratorio de Biofísica y Biología Molecular, Departamento de Bioquímica y Biología Molecular, Facultad de Ciencias, Universidad Autónoma del Estado de Morelos, Cuernavaca, Morelos, 62210, Mexico; 2 Instituto de Agrobiotecnología, IdAB-CSIC-Universidad Pública de Navarra-Gobierno de Navarra, Navarre, E-31192, Spain; 3 Grain, Forage and Bioenergy Research Unit, USDA-ARS, University of Nebraska-Lincoln, Lincoln, NE, 68583-0937, USA

**Keywords:** Evolution, function, gene expression, non-symbiotic, structure, symbiotic, truncated

## Abstract

Hemoglobins (Hbs) corresponding to non-symbiotic (nsHb) and truncated (tHb) Hbs have been identified in rice ( *Oryza*). This review discusses the major findings from the current studies on rice Hbs. At the molecular level, a family of the *nshb* genes, consisting of *hb1*, *hb2*, *hb3*, *hb4* and *hb5*, and a single copy of the *thb* gene exist in *Oryza sativa* var. indica and *O*. *sativa *var. japonica, Hb transcripts coexist in rice organs and Hb polypeptides exist in rice embryonic and vegetative organs and in the cytoplasm of differentiating cells. At the structural level, the crystal structure of rice Hb1 has been elucidated, and the structures of the other rice Hbs have been modeled. Kinetic analysis indicated that rice Hb1 and 2, and possibly rice Hb3 and 4, exhibit a very high affinity for O _2_, whereas rice Hb5 and tHb possibly exhibit a low to moderate affinity for O _2_. Based on the accumulated information on the properties of rice Hbs and data from the analysis of other plant and non-plant Hbs, it is likely that Hbs play a variety of roles in rice organs, including O _2_-transport, O _2_-sensing, NO-scavenging and redox-signaling. From an evolutionary perspective, an outline for the evolution of rice Hbs is available. Rice *nshb* and *thb* genes vertically evolved through different lineages, rice nsHbs evolved into clade I and clade II lineages and rice *nshb*s and *thb*s evolved under the effect of neutral selection. This review also reveals lacunae in our ability to completely understand rice Hbs. Primary lacunae are the absence of experimental information about the precise functions of rice Hbs, the properties of modeled rice Hbs and the *cis*-elements and *trans*-acting factors that regulate the expression of rice *hb* genes, and the partial understanding of the evolution of rice Hbs.

## Abbreviations

2,4-D, 2,4-dichlorophenoxyacetic acid; ARR1, Arabidopsis response regulator 1; BCIP, 5-bromo-4-chloro-3´-indolyphosphate; Hb, hemoglobin; Lb, leghemoglobin; MIP1, macrophage inflammatory protein 1; mya, million of years ago; NBT, nitro-blue tetrazolium; nsHb, non-symbiotic hemoglobin; nsHb-1, non-symbiotic hemoglobin type 1; nsHb-2, non-symbiotic hemoglobin type 2; nsHb-I, clade I non-symbiotic hemoglobin; nsHb-II, clade II non-symbiotic hemoglobin; RT-PCR, reverse transcriptase-polymerase chain reaction; SNP, sodium nitroprusside; tHb, truncated (2/2) hemoglobin.

## Introduction

Two decades ago Taylor and co-workers reported the cloning and sequencing of a hemoglobin (Hb) cDNA from barley ^1^. This was the first report about the existence of Hbs in monocotyledonous plants. Since then Hbs have been identified in a number of monocots, including rice ^2^, maize ^3^ and wheat ^4^. Rice Hbs and genes coding for these proteins are rather well characterized, thus in some aspects rice Hbs are a model to understand monocot and other land plant Hbs. However, the accumulated information on rice Hbs over the last seventeen years is scattered. This review discusses major findings from the study of rice Hbs including a historical perspective, and proposes biochemical and physiological mechanisms for rice Hbs based on information available about rice Hbs and other monocot and land plant Hbs. For general aspects and the biochemistry, physiology and evolution of plant Hbs, we recommend to the reader reviews published elsewhere ^5– 15^.

### Generalities on hemoglobins

Hb is known to the reader because this protein is responsible for the red color of vertebrates´ blood ^16^. However, Hbs are widely distributed in living organisms, ranging from bacteria to mammals ^17, 18^. The tertiary structure of Hbs consists of a specific arrangement of 6 to 8 α-helices (designated with letters A to H) known as the globin-fold. This protein folding forms a hydrophobic pocket where a heme prosthetic group is located ^16, 19^. Two structural types of the globin-fold have been identified in Hbs: the 2/2- and 3/3-folding. In the 2/2-Hbs, helices B and E overlap to helices G and H and in the 3/3-Hbs helices A, E and F overlap to helices B, G and H. Likewise, three evolutionary families have been identified in Hbs: the M, S and T Hb families. The M Hbs, which exist in bacteria and eukaryotes, include flavoHbs and single domain globins, the S Hbs, which exist in bacteria and some fungi, include globin-coupled sensors, protoglobins and single domain globin sensors, and the T Hbs, which exist in bacteria, unicellular eukaryotes and plants, include truncated Hbs (tHbs). Canonical T Hbs from bacteria and unicellular eukaryotes are ~100 to 120 amino acids in length, however plant T Hbs are longer than canonical T Hbs because of the existence of extra amino acids at the N- and C-terminal. The M and S Hbs fold into the 3/3-folding whereas the T Hbs fold into the 2/2-folding ^18, 20– 25^.

A variety of ligands bind to the heme iron of Hbs, including O _2_ and NO. Reversible binding of O _2_ is closely associated to the major function of Hbs in organisms, which is the transport of O _2_^16^. Binding of NO by oxygenated Hbs is essential to NO-detoxification via a NO-dioxygenase activity ^26^. Several additional functions have been reported for Hbs, including dehaloperoxidase activity and reaction with free radicals, binding and transport of sulfide and lipids, and O _2_-sensing ^27– 32^. This indicates that *in vivo* Hbs might be multifunctional proteins.

### Land plant hemoglobins

Land plant Hbs were first identified by Kubo in soybean root nodules ^33^. Few years after Kubo´s discovery these proteins were named as leghemoglobins (Lbs) by Virtanen and Lane ^34^ because they were only found in the symbiotic (N _2_-fixing-) nodules of the leguminous plants. Lbs are the most abundant soluble proteins in nodules ( *e.g*. in soybean nodules their concentration is as high as 3 mM) ^14, 35^. The x-ray analysis of lupin Lb revealed that the tertiary structure of Lbs was remarkably similar to that of the sperm whale myoglobin ^36^. This evidence demonstrated that Lbs are plant Hbs and indicated that plant and animal Hbs evolved from a common ancestor more than 600 mya ^6^. Subsequent work led to the identification of Lb-like (or symbiotic) Hbs in nodules of actinorhizal plants ^37– 41^, purification of an Hb from the root nodules of the dicotyledonous non-legume *Parasponia andersonii* ^42^, cloning and sequencing of an *hb* gene from the non-nodulating dicot *Trema tomentosa* ^43, 44^ and detection of Hbs in non-symbiotic organs from several land plants, including primitive bryophytes and evolved angiosperms ^9, 15, 45– 47^. Until now three types of Hbs have been identified in land plants: the symbiotic Hbs, which include Lbs, that are specifically located within nodules of the N _2_-fixing land plants, and the non-symbiotic (nsHbs) and truncated (tHbs) Hbs, that are located within non-symbiotic and symbiotic organs of primitive and evolved land plants ^9, 15^. Based on sequence similarity the nsHbs are further classified into type 1 and type 2 nsHbs (nsHbs-1 and nsHbs-2, respectively) ^9, 48, 49^.

### Distribution of hemoglobins in monocotyledonous plants

Monocots are a large family of flowering plants ^50^ that includes cereals. Cereals, such as rice, maize and wheat, are the main source of food for humans. Because of this, during that last decade the genomes of a number of cereals have been sequenced. This allowed the identification of novel cereal Hbs. The search of *hb* genes in databases by G. Rodríguez-Alonso and R. Arredondo-Peter ^51, 52^ revealed that nsHb and tHb sequences exist in the *Brachypodium distachyon*, *Hordeum vulgare* (barley), *Oryza glaberrima* (rice), *O*. *rufipogon* (rice), *O*. *sativa* (rice) var. indica, *O*. *sativa* (rice) var. japonica, *Panicum virgatum* (switchgrass), *Setaria italica* (foxtail millet), *Sorghum bicolor* (sorghum), *Triticum aestivum* (wheat) and *Zea mays* ssp. *mays* (maize) genomes. The highest number of nsHbs (5) exists in *O*. *sativa* var. indica and *O*. *sativa* var. japonica, whereas one to three nsHbs exist in barley, *Brachypodium*, foxtail millet, maize, *O*. *glaberrima*, *O*. *rufipogon*, sorghum, switchgrass and wheat. Also, with the exception of wheat, which contains two copies of the *thb* gene, a single copy of *thb* was identified in the genome of *Brachypodium*, barley, *O*. *sativa* var. indica, *O*. *sativa* var. japonica, switchgrass, foxtail millet, sorghum and maize. Little is known about Hbs from non-cultivated monocots. The only Hb reported from a non-cultivated monocot is that of teosinte ( *Z*. *mays* ssp. *parviglumis*) ^3^, which is postulated as the ancestor of maize ^53, 54^. Analysis by Southern blot using the teosinte *hb* gene as probe showed that apparently a single copy of *hb* exists in teosinte (J. Sáenz-Rivera and R. Arredondo-Peter, unpublished results). Sequence comparison revealed that maize and teosinte Hb polypeptides are identical ^3^.

### Early search and identification of rice hemoglobins

**Figure 1.**
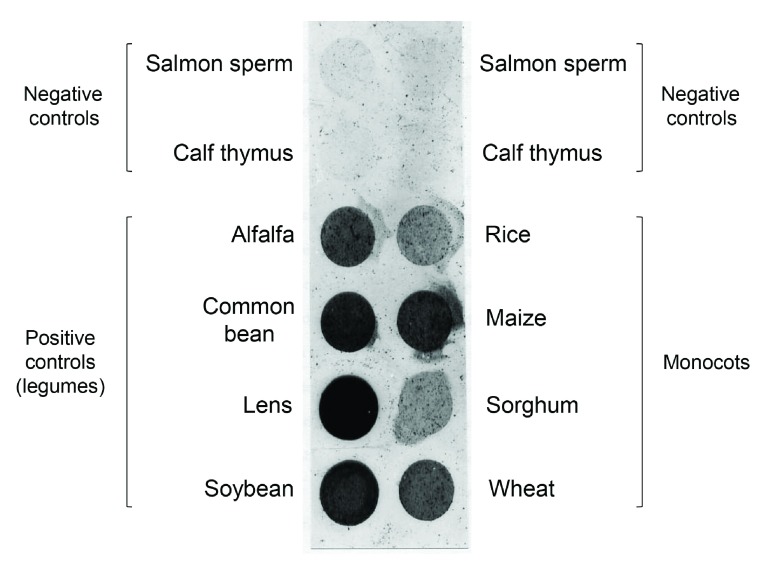
Early (1991) detection of rice, maize, sorghum and wheat *hb*-like sequences by dot-blot hybridization (R. Arredondo-Peter, unpublished results). Approximately 20 μg of undigested total DNA was used as template and a consensus oligonucleotide for legume and non-legume plant Hbs ^58^ was used as probe. Sequence of the consensus probe was 5´-GTA GCC TAT GAT GAA TTG GCA GCT GCA ATT AAG-3´. The probe was labeled by nick translation with Biotin-dATP using a Bionick labeling system (Gibco BRL). The membrane was prehybridized with SSC 2× for 4h at 42°C, hybridized overnight at the same temperature, washed at high stringency (SSC 2×/SDS 0.1% for 3 min at room temperature, SSC 0.2×/SDS 0.1% for 15 min at room temperature and SSC 0.16×/SDS 0.1% for 15 min at 65°C) and incubated with the streptavidin-alkaline phosphatase conjugate and the BCIP/NBT mix to develop color. Animal (salmon sperm and calf thymus) and legume DNAs were included as negative and positive controls, respectively.

Monocots were a target for searching Hbs after these proteins were detected in non-symbiotic organs of dicotyledonous plants (see subsection above). At that time, monocot genomes had not been sequenced. Searching approaches consisted in detecting Hb polypeptides and *hb* genes by spectroscopy and molecular biology methods, respectively. Attempts to detect absorption maxima in the Soret (~410 nm) and Q (~500 to 550 nm) regions, which are characteristic of ferric (Fe ^3+^), ferrous (Fe ^2+^) and liganded Hbs ^55, 56^, were unsuccessful (R. V. Klucas and C. A. Appleby, unpublished results) mostly due to the very low Hb concentration (~50 to 100 nM) in plant non-symbiotic organs ^5, 57^. At the molecular level a consensus probe designed from legume and non-legume ( *T*. *tomentosa*, *P*. *andersonii* and *Casuarina glauca*) Hb sequences ^58^ hybridized with *hb*-like sequences from rice and other monocot total DNAs ( Figure 1). This observation suggested that *hb* sequences exist in monocots, however hybridizing fragments were not subsequently cloned and sequenced in order to verify if they actually corresponded to *hb* genes.

Rice Expressed Sequence Tags (ESTs) were first deposited in databases early in the 1990´s. The first rice Hb (Hb1 and Hb2) sequences were detected from ESTs deposited in the DNA Data Bank of Japan (DDBJ) database ^59^. Rice Hb1 and Hb2 corresponded to clones C741 and C2576 with DDBJ accession number D15507 and D38931, respectively. Rice *hb1* and *hb2* genes were subsequently amplified by PCR, cloned and sequenced. Sequence analysis revealed that rice *hb1* codes for non-symbiotic Hb1 and that rice *hb2* codes for non-symbiotic Hb2 ^2^. Afterwards, sequencing of the rice ( *O*. *sativa* L. ssp. *indica*) genome more than a decade ago ^60^ allowed the identification of a family of rice *nshb* genes and a single copy of the rice *thb* gene (see subsection below).

## Molecular biology of rice hemoglobins

### Rice hemoglobin genes

The *O*. *sativa* var. indica and *O*. *sativa* var. japonica genomes are fully sequenced, and the *O*. *glaberrima* and *O*. *rufipogon* genomes are partially sequenced. Rice genome sequences are mainly available from the GenBank ( www.ncbi.nlm.nih.gov) and Phytozome ( http://www.phytozome.org/) databases. Search of Hb sequences in the above databases showed that a family of the *nshb* genes, consisting of *hb1*, *hb2*, *hb3*, *hb4* and *hb5*, and a single copy of the *thb* gene exist in the *O*. *sativa* var. indica and *O*. *sativa* var. japonica genomes. A single copy of the *nshb* gene was detected in the *O*. *glaberrima* and *O*. *rufipogon* genomes, however *thb* genes have not yet been detected in these plants ^52^. Given that the sequencing of the *O*. *glaberrima* and *O*. *rufipogon* genomes is in progress the identification of *hb* genes in these genomes is incomplete. Thus, the following discussion will focus on the *O*. *sativa* var. indica and *O*. *sativa* var. japonica *hb*s. However, we must clarify to the reader that the sequence of Hb1, Hb2, Hb3, Hb4 and tHb and Hb5 polypeptides are 100% and 97% identical between *O*. *sativa* var. indica and *O*. *sativa* var. japonica, respectively. Therefore, the subsequent discussion on the *O*. *sativa* Hbs will indistinctively correspond to either *O*. *sativa* var. indica or *O*. *sativa* var. japonica.

**Figure 2.**
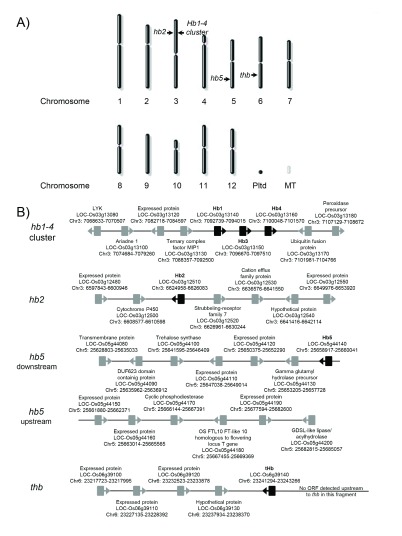
Localization of *hb*s in the *O*. *sativa* chromosomes (A) and mapping of *hb* genes into the *O*. *sativa* genome (B). The *hb* genes were localized in the rice chromosomes by BlastN analysis using the rice ( *O*. *sativa*) genome resource from the GenBank database as template and the sequence for the rice *hb1*, *hb3* and *hb4* (GenBank accession number AF335504), *hb2* and *hb5* (GenBank accession numbers AF335503 and EF061459, respectively) and *thb* (GenBank accession number NM_001064507) genes as probes. The *hb* (black boxes) and flanking (gray boxes) genes were mapped into 50 kb fragments of the *O*. *sativa* genome by BlastN2.2.26+ analysis using the Phytozome V9.1 server ( www.phytozome.org) and the above *hb* sequences as probes. Arrows indicate the transcription orientation. Information for each gene corresponds to predicted protein (following the Phytozome nomenclature), locus name in the *O*. *sativa* genome and position at the *O*. *sativa* chromosome. Gene sizes and distance between genes are not shown at scale. Pltd, chloroplast chromosome; MT, mitochondrial chromosome.

The structure of known rice *hb* genes corresponds to four exons and three introns, with introns located at similar position as all of the known plant *hb* genes ^61^. Canonical TATA boxes and a variety of potential promoters exist upstream of the rice *hb* genes which suggests that rice *hb*s are functional and that the regulation of the *hb* genes in this plant is complex ^62– 64^. Figure 2 shows the localization of *hb*s in the *O*. *sativa* chromosomes and mapping of *hb*s in the *O*. *sativa* genome. Rice *hb1*, *hb3* and *hb4* cluster forming the *hb1*- *hb4* cluster ^63^ which is localized in chromosome 3. Rice *hb2* is also localized in chromosome 3 but 467 kb upstream of the *hb1*- *hb4* cluster. In contrast, rice *hb5* and *thb* genes are localized in chromosomes 5 and 6, respectively ( Figure 2A). Rice *hb*s are flanked by a variety of genes with known and unidentified functions ( Figure 2B). However, with the exception of genes coding for a ternary complex factor macrophage inflammatory protein MIP1 and an ubiquitin fusion protein which are located 239 and 411 nucleotides up- and downstream of the *hb1*- *hb4* cluster, respectively, distance of flanking genes to *hb*s is >1 kb. This suggests that co-expression of *hb* and flanking genes is unlikely.

### Gene expression and localization of hemoglobins in rice organs

The expression of *hb* genes and localization of Hb polypeptides have been analyzed in rice growing under normal and stressed conditions. Under normal conditions the expression level of rice *nshb*s was low ^2, 62^. However, analysis by RT-PCR revealed that *hb1*, *hb2* and *hb5* genes were expressed in embryonic and vegetative organs obtained from rice plants grown under a normal environment ^2, 62, 65^. Specifically, transcripts for rice Hb1 were detected in embryos, seminal roots, leaves and roots, transcripts for rice Hb2 were detected in embryos, coleoptiles, seminal roots and leaves, and transcripts for rice Hb5 were detected in embryos, coleoptiles, seminal roots, leaves and roots. Likewise, evaluation of the β-glucuronidase (GUS) activity from a construct containing the rice *nshb2* gene promoter that is responsive to the cytokinin-regulated ARR1 *trans*-acting factor showed that this promoter is activated in roots, the vasculature of young leaves, flowers and the pedicel/stem junction of transgenic *Arabidopsis* ^64^. In addition, a variety of potential promoters was identified upstream of the rice *nshb* genes, such as those involved in the ethylene synthesis, photoregulation, heat shock response and plant defense signaling ^57, 62– 64^. However the activities of these promoters have not been determined.

Transcriptomic analyses revealed that nsHb and tHb transcripts coexist in rice embryonic and vegetative organs ( Table 1). This evidence suggests that nsHb ( *i.*e *.* Hb1, Hb2, Hb3, Hb4 and Hb5) and tHb polypeptides coexist and probably function in rice organs. Immunoanalysis by Western blot and confocal microscopy using a polyclonal anti-rice Hb1 antibody revealed that Hb polypeptides exist in rice seeds and in rice leaves and roots from 2 to 14 weeks after seed germination. These analyses also revealed that Hb polypeptides exist in the cytoplasm of differentiating cells of the root cap, schlerenchyma, aleurone, and in the vasculature, principally in the differentiating xylem ^14, 57, 66^. However, the anti-rice Hb1 antibodies cross-react with different rice Hbs (G. Sarath and E. J. H. Ross, unpublished results) and thus it is not known which Hb polypeptides were detected in the above analyses by the anti-rice Hb1 antibodies.

**Table 1.** Detection of Hb transcripts in organs from rice growing under normal and (cold, drought and salt) stressed conditions. Rice Hb transcripts were detected in plant organs using The Rice Genome Annotation Project database ( http://rice.plantbiology.msu.edu/) and hemoglobin as keyword (S. Castro-Bustos and R. Arredondo-Peter, unpublished).

Rice organs	Hb transcripts					
	Hb1	Hb2	Hb3	Hb4	Hb5	tHb
**Normal conditions**						
Seed						
Endosperm	✓	✓	✓		✓	✓
Embryo	✓	✓	✓	✓	✓	✓
Vegetative rice						
Leaves	✓	✓	✓		✓	✓
Stems	✓		✓	✓	✓	
Roots	✓	✓	✓	✓	✓	
Reproductive rice						
Inflorescence		✓		✓	✓	✓
Leaves	✓	✓	✓		✓	
Stems	✓	✓	✓	✓	✓	
Roots	✓	✓	✓	✓	✓	
Reproductive organs						
Lemma					✓	
Anther	✓	✓			✓	✓
Palea			✓		✓	
Ovary	✓	✓	✓	✓	✓	
Pistil	✓	✓	✓		✓	✓
**Stress conditions**						
Reported as part of the plant response to stress	✓	✓	✓	✓	✓	✓

It is well documented that land plant *hb* genes are either up- or down-regulated by stress conditions ^1, 45, 66– 69^. Table 1 shows that Hb transcripts coexist in rice growing under cold, drought and salt stress conditions. Also, Ohwaki and co-workers ^70^ reported that *nshb1* and *nshb2* are induced by nitrate, nitrite and NO in cultured rice cells. These observations indicate that rice *hb* genes response to a variety of stress conditions. However, the detection of Hb polypeptides by Western blot using the anti-rice Hb1 antibodies showed that level of Hbs increased in rice etiolated leaves and flooded roots, but not in rice plants subjected to oxidative (H _2_O _2_), nitrosative (SNP) and hormonal (2,4-D) stresses. These observations suggest that rice Hbs do not appear to be part of a generalized stress response, but may be functional in plant organs subjected to specific stress conditions ^66^.

## Structure and biophysical properties of rice hemoglobins

### Structure of rice non-symbiotic hemoglobins

**Figure 3.**
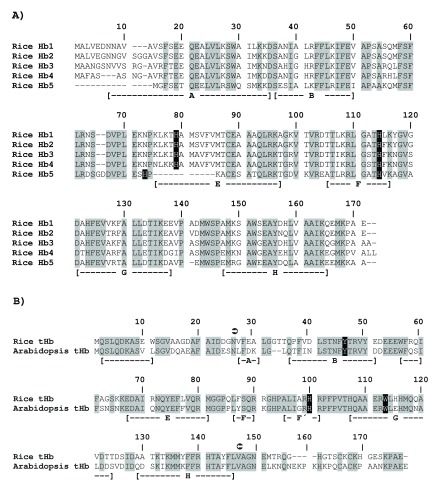
Sequence alignment of rice Hbs. ( **A**) Sequence alignment of rice nsHbs. Note the 11 amino acids deletion in rice Hb5 at position 75–85. Modified from Garrocho-Villegas *et al*. ^62^. ( **B**) Sequence alignment of rice and *Arabidopsis* (GenBank accession number AAK55409) tHbs. Distal and proximal His in rice nsHbs (H79 and H114, respectively) and proximal His (H100) and proposed distal Tyr/Tre (Y46/W113) in *Arabidopsis* ^86^ and rice tHbs and conserved amino acids are shown with black and gray background, respectively. Helices are indicated with letters A to H based on the crystal structure of rice Hb1 ^71^ in rice nsHbs and on the crystal structure of *Arabidopsis* tHb ^86^ in rice tHb. Left- and right-oriented arrows within a black circle in the rice and *Arabidopsis* tHbs sequence alignment delimit the globin domain.

Rice *hb* genes are functional and code for Hb polypeptides with a predicted molecular mass of ~16 to 19 kDa. Also, sequences among rice nsHb polypeptides are highly similar: Hb1 and Hb2 are 93% similar to each other, Hb3 and Hb4 are 87.1% similar to each other, and 85.5% and 84.7%, and 79.2% and 82.2% similar to Hb1 and Hb2, respectively, and rice Hb1 and Hb5 are 67% similar to each other ^2, 62, 63^ ( Figure 3A).

**Figure 4.**
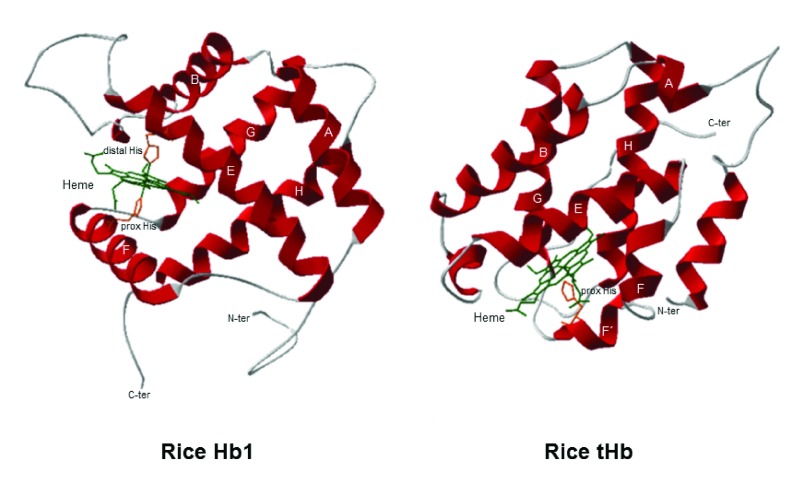
Crystal structure of rice Hb1 (PDB ID 1D8U) and predicted structure of rice tHb. The tertiary structure of rice tHb was modeled using the automated mode of the I-Tasser server ( http://zhanglab.ccmb.med.umich.edu/I-TASSER/) ^131– 133^ and the crystal structure of the *Thermobifida fusca* tHb (PDB ID 2BMM) as the structural homologue. Model for the rice tHb is deposited in the Caspur Protein Model Database ( http://bioinformatics.cineca.it/PMDB/main.php) under the ID number PM0079484. Helices are indicated with letters A to H. Note the overlapping of helices A, E and F to helices B, G and H in (3/3-folding) rice Hb1, and overlapping of helices B and E to helices G and H in (2/2-folding) rice tHb. Heme prosthetic group is shown in dark green color and proximal and distal His are shown in light brown color.

Rice Hb1 was the first monocot nsHb whose crystal structure was elucidated ^71^. This protein crystalizes as a dimer when its concentration is ≥1 mM ^72^. After the elucidation of the rice Hb1 structure the tertiary structure of rice Hb2 ^73^, Hb3, Hb4 and Hb5 ^62^ (CASPUR PMDB ID PM0075009, PM0075873, PM0076005 and PM0075011, respectively) was predicted using computational methods and rice Hb1 (PDB ID 1D8U) as the structural homolog. The crystal structure of rice Hb1 and that of predicted rice Hb2, Hb3 and Hb4 is highly similar. The tertiary structure of these proteins consists of six helices that fold into the 3/3-folding (see subsection on *Generalities on hemoglobins*). However, the structure of rice Hb1 to 4 is characterized by the existence of a short pre-helix A located at the N-terminal and an extended and poorly ordered CD-loop. The heme pocket in these proteins differs from that in “traditional” Hbs because the proximal and distal His side chains coordinate the heme iron forming a hemichrome ( Figure 4), resulting in that heme iron from rice Hb1 to 4 is hexacoordinate. Also, the amino acid residues (V50, S53, E125, V126, F129 and A130 from Figure 3A) located at the monomer-monomer interface of dimeric rice Hb1 ^71^ are highly conserved in rice Hb2 to 4 ^63^. This suggests that rice Hb1 to 4 can potentially form homo- or hetero-dimers if the *hb1* to *4* genes coexpress in rice organs. The tertiary structure of rice Hb5 also consists of six helices that fold into the 3/3-folding. However, rice Hb5 differs from rice Hb1 to 4 in missing 11 amino acids in helix E ( Figure 3A) which results in that the length of the CD-loop and helix E in the predicted Hb5 structure are unusually long and short, respectively. An apparent consequence from this characteristic is that distal His is located far away (13.92 Å, compared to 2.11 Å in rice Hb1) from the heme iron within the predicted Hb5 structure, resulting in that heme iron from rice Hb5 could be pentacoordinate ^62^. The amino acid residues located at the monomer-monomer interface of dimeric rice Hb1 ^71^ are poorly conserved in rice Hb5 ^62^ ( Figure 3A) which suggests that rice Hb5 exists *in vivo* as a monomer.

The folding pathway and kinetics of rice nsHbs were predicted using the Average Distance Map (ADM) method ^74– 76^. This analysis indicated that rice Hb1 and Hb2 could fold in the C → N direction at a moderate rate, that rice Hb3 could fold in the N → C direction at a fast rate, and that rice Hb4 and Hb5 could fold in the N → C direction at a moderate rate. Thus, it appears that the predicted folding pathway and kinetics among rice nsHbs are diverse. Also, the ADM analysis showed that pre-helix A and CD-loop apparently do not play a role during the folding of rice nsHbs ^77^. The physiological relevance of the folding pathways for rice nsHbs, including the polypeptide association with the heme, is still not known.

### Spectroscopic characteristics of rice non-symbiotic hemoglobins

Visible spectroscopy (see subsection *Early search and identification of rice hemoglobins*) is a tool to analyze the redox state of and ligand-binding to the heme iron of Hbs ^56, 78, 79^. Rice Hb1 is the only rice nsHb that has been spectroscopically characterized ^2^. This protein exhibits spectral characteristics that are similar to other Hbs. However, rice Hb1 exhibits distinctive absorption maxima in the deoxyferrous form: the unligated ferrous state exhibits maxima at 526 and 556 nm ^2^ which are characteristic of hexacoordinate heme iron ^80^. This is in contrast to pentacoordinate Hbs which display a broad peak centered at 556 nm in their deoxyferrous form ^7, 81, 82^. The distal ligand that coordinates the heme iron in rice Hb1 was identified as His74 by site directed mutagenesis. Absorbance spectra of the ferric and deoxyferrous forms of an H74L mutant of rice Hb1 showed no evidence of His coordination. Also, the addition of exogenous imidazole to ferric and deoxyferrous H74L mutant resulted in a spectrum identical to that of the wild-type rice Hb1 ^2^. This evidence indicated that in rice Hb1 the distal ligand to heme iron is His74. A similar case can be predicted for rice Hb2 to 4. In contrast, distal His appears to be located far away from the heme iron in the predicted structure of deoxyferrous rice Hb5, resulting in that heme iron in rice Hb5 could be pentacoordinate ^62^.

### Rate and equilibrium constants for the reaction of oxygen from rice non-symbiotic hemoglobins

Analysis of ligand-association and -dissociation rate constants of penta- and hexacoordinate Hbs using stopped-flow methods indicated that these proteins exhibit low to moderate and high affinity for O _2_, respectively. Rice Hb1 is hexacoordinate and apparently rice Hb2 to 4 are hexacoordinate and rice Hb5 is pentacoordinate. The O _2_-association rate constants for rice Hb1 and 2, and possibly for rice Hb3 and 4, are rather similar to those of other O _2_-transport and -storage proteins, such as the sperm whale myoglobin and soybean Lb *a* ( Table 2). However, in rice Hb1 ^2^ and 2 ^14^, and possibly in rice Hb3 and 4, the bound O _2_ is stabilized by distal His after binding to the heme iron, which results in very low O _2_-dissociation rate constants. The O _2_-association and -dissociation rate constants of hexacoordinate rice Hb1 and 2, and possibly of rice Hb3 and 4, result in that the affinity of these proteins for O _2_ is very high ( Table 2). In the absence of biochemical data it becomes difficult to evaluate the O _2_-binding characteristics of rice Hb5, however, its predicted pentacoordinate structure would suggest a low to moderate affinity for O _2_.

**Table 2.** Rate and equilibrium constants for the reaction of O _2_ from rice Hb1 and 2. Constants for arbitrarily selected plant and non-plant Hbs are included for comparison.

Protein	*k*´O _2_ (μM ^-1^s ^-1^)	*k*O _2_ (s ^-1^)	*K*O _2_ (μM ^-1^)	Reference
**Plant Hbs**				
nsHbs				
Rice Hb1	68	0.038	1800	2
Rice Hb2	50	0.038	1316	14
Barley Hb	2.4	0.028	86	134
*Arabidopsis* AtGLB1	74	0.12	617	49
*Arabidopsis* AtGLB2	1	0.17	7	49
*Lotus* Glb1-1	81	0.004	20,250	111
*Lotus* Glb1-2	300	0.27	1111	111
tHbs				
*Arabidopsis* AtGLB3	0.2	0.3	0.66	69
Symbiotic Hbs				
Soybean Lb *a*	130	5.6	23	135
**Non-plant Hbs**				
Sperm whale myoglobin	14	11	1.3	136
*Ascaris* Hb	1.5	0.004	375	137
*Paramphistomum* Hb	108	0.033	3270	137
*Synecocystis* tHb	240	0.011	21,818	138

*k*´O _2_ is the O _2_-association rate constant; *k*O _2_ is the O _2_-dissociation rate constant; *K*O _2_ ( *k*´O _2_/ *k*O _2_) is the O _2_-affinity constant.

### Postulated migration routes for gaseous ligands to the heme iron in rice Hb1

The bis-histidyl hexacoordinated form of rice Hb1 displays a hydrophobic distal cavity which appears to be connected with the external solvent through the position of Phe44 (also known as FB10 because it occupies the tenth position in helix B). It was suggested that this amino acid regulates the migration of small ligands in rice Hb1, for example in ligand binding to the heme iron, ligand migration through internal docking sites and ligand release into the external solvent ^83, 84^. Kinetic analysis after laser flash photolysis of rice Hb1 encapsulated in silica gel combined with computational analysis revealed the existence of two channels in the rice Hb1 CO-bound species. The first channel is located in the distal region of the heme pocket and is connected with a secondary channel that is directly connected with the external solvent. Apparently, the position of FB10 in hexacoordinated rice Hb1 leaves the distal heme pocket accessible to the external solvent, however after the ligand entrance the phenyl ring rotates closing the cavity and thus hindering the exit of the bound ligand ^85^. Thus, together with distal His (see subsection *Spectroscopic characteristics of rice non-symbiotic hemoglobins*) and aromatic amino acids that are located in the distal region of the heme pocket, FB10 appears to regulate hexacoordination and functioning of rice Hb1.

### Rice truncated hemoglobin, predicted structure and properties

Rice ( *O*. *sativa*) tHb (GenBank accession number NP_001057972) is 172 amino acids in length, which corresponds to a globin domain (position 26 to 147) flanked by N- and C-terminal extensions ( Figure 3B). No monocot tHb has been analyzed by x-ray crystallography, however the tertiary structure of a rice tHb was predicted using computational methods ( Figure 4). The predicted structure of rice tHb is highly similar to the crystal structure of an *Arabidopsis thaliana* tHb ^86^. The globin domain from rice and *A*. *thaliana* tHbs folds into the 2/2-folding (see subsection on *Generalities on hemoglobins*). Similarly to the *A*. *thaliana* tHb structure, flanking regions to the globin domain of predicted rice tHb correspond to an N-terminal helical extension and a C-terminal unfolded extension ( Figure 4). The high similarity between the crystal structure of *A*. *thaliana* tHb and the predicted structure of rice tHb suggests that the biochemical properties and function of dicot and monocot tHbs are similar.

Rice tHb has not been subjected to spectral analysis, however the predicted structure of this protein ( Figure 4) is highly similar to the crystal structure of an *A*. *thaliana* tHb ^86^ (see above). The absorption spectra of an *A*. *thaliana* tHb showed that heme iron from this protein is pentacoordinate ^69, 86^. Thus, it is likely that heme iron in rice tHb is pentacoordinate and that the rate and equilibrium constants for the reaction of O _2_ of rice tHb are similar to those of the *Arabidopsis* tHb ( Table 2), *i.e*. the O _2_-association and -dissociation rate constants are low to moderate.

## Postulated functions for rice hemoglobins

While data on the localization, kinetics, regulation and structure of rice Hbs have accumulated, little work has been performed to fully understand the function of these proteins in rice organs. However, previous work from other plant and non-plant Hbs provides data that enable us to propose potential functions for rice Hbs. Rice Hbs could potentially function within cells through O _2_-transport and -signaling, binding to small molecules (most notably NO) and other as yet undetermined mechanisms. Here we evaluate the evidence for and against these modes of action.

Oxygen transport is a major function of many Hbs. This process requires that the kinetics of O _2_-binding do not limit the O _2_-diffusion process ^87– 90^. Based on the concentration of Hb polypeptides in rice organs (~50 to 100 nM) ^57^, the O _2_-association rate constant of rice Hb1 and 2 ( Table 2) and possibly that of rice Hb3 to 5 and tHb (see subsections *Rate and equilibrium constants for the reaction of oxygen from rice non-symbiotic hemoglobins* and *Rice truncated hemoglobin, predicted structure and properties*), and the free O _2_ concentration in aerated rice roots (<1.4 μM) ^91^, it is likely that Hbs would be substantially oxygenated in rice organs. However, the O _2_-dissociation rate constants of rice Hb1 and 2 ( Table 2), and possibly that of rice Hb3 and 4, are extremely low. These data do not support the O _2_-transport function for rice Hb1 to 4 because these proteins would not release O _2_ after oxygenation.

It was reported that hexacoordinate Hbs interact with either organic molecules or protein partners ^27, 92^ and thus a possibility is that such interactions could impact the kinetic constants, particularly the O _2_-dissociation rate constants, of hexacoordinate nsHbs ^93^. There have been no direct biochemical evaluations of this hypothesis in rice or in other plants, precluding definitive answers. However, their unique structural features could result in as yet undiscovered interactions.

Rice Hbs may function in O _2_-signaling if they easily bind and release O _2_. Appleby and co-workers ^5^ proposed that under normal conditions Hbs would be oxygenated and under O _2_-limiting conditions the concentration of deoxyHb would increase triggering an anaerobic response. It was reported that levels of Hbs increase in rice roots from flooded plants indicating that the synthesis of rice Hbs increases under O _2_-limiting conditions ^66^. Rice is a flooding resistant crop, thus under flooding ( *i.e*. hypoxia) conditions rice Hbs could sense low O _2_-concentrations and trigger an anaerobic metabolism for rice growth. To act as a signaling molecule, rice Hbs will need to bind directly to the DNA, to additional proteins, such as transcription factors, or catalyze some unique reactions that can influence key downstream events. To date there are no reports of immunoprecipitation experiments specially targeting rice Hbs coupled to further proteomic analysis. It is thus uncertain if rice Hbs bind to other partners. There is also no structural evidence that indicates that rice Hbs can bind directly to DNA. *In planta*, they appear to be soluble and essentially contained within the cytoplasm ^57^. There are reports of nuclear-localized Hbs ^94^, but no direct evidence for a function arising from translocation of Hbs from the cytoplasm to the nucleus currently exist.

The NO dioxygenase activity exhibited by oxygenated Hbs is well documented ^95– 97^. NO is a hormone-like radical that modulates several aspects of the plant physiology, including plant immunity, seed germination, de-etiolation, apoptosis, stomata guard cells opening/closure and the rhizobia-legume symbiosis ^98– 100^. Scavenging of NO is considered a function of plant Hbs ^10, 101– 104^. During this process, oxygenated plant Hbs react with NO producing nitrate and ferric Hb. Ferric plant Hbs are subsequently reduced to ferrous Hb by enzymatic ^105, 106^ and non-enzymatic ^107– 111^ mechanisms. This process regenerates (oxy) ferrous Hb which is able to bind NO in a cyclic pathway referred to as the Hb/NO cycle ^104, 112^. The operation of this cycle appears to be involved in maintaining an active metabolism in the plant cells ^10^. Rice Hb1 exhibits NO dioxygenase activity (k _obs, NOD_ = 90 s ^-1^) ^113^ thus a possible function of Hbs into the rice physiology is modulating levels of NO by scavenging NO. However, the inability of rice Hb1 to substitute the NO scavenger activity in a flavoHb knockout *Escherichia coli* ^113^ and the observation that levels of Hbs did not change in rice seeds germinated under nitrosative stress ^66^ suggest that the NO dioxygenase activity of rice Hbs is limited *in vivo*.

A consequence of the operation of the Hb/NO cycle could be the maintenance of cell respiration and energy status. Based on the studies on over- and under-expressing barley nsHb in maize cells, it was proposed that under hypoxic conditions barley nsHb is involved in the ATP metabolism, particularly in maintaining the energy status under O _2_-limiting conditions ^114^. Immunolocalization data showed that rice Hbs are localized in differentiating cells (see subsection on *Gene expression and localization of hemoglobins in rice organs*) ^57^. The metabolism of these cells is redirected in response to differentiation signals, such as a change in the cell redox state. Rice Hbs could be involved in redox signaling if the redox state of the heme is functional ^71^. Thus, under these conditions rice Hbs may function by sensing or maintaining redox environments that promote specific cell metabolisms ^14^.

It was proposed that one of the functions of plant Hbs could be related to the peroxidase activity ^8, 93^. This is of interest because peroxidase activity modulates the levels of reactive oxygen species and a variety of cellular processes ^115– 121^. In plants, evaluation of the peroxidase activities of *Arabidopsis* Hbs (AtGLB1, AtGLB2 and AtGLB3) revealed that these proteins oxidize Amplex Red, DHR123 and guaiacol substrates ^122^ and overexpression of AtGLB1 increased tolerance of *Arabidopsis* to H _2_O _2_ stress ^123^. These observations suggested that *Arabidopsis* Hbs function as antoxidants. However, levels of Hb polypeptides did not change in rice seeds germinated under H _2_O _2_ stress ^66^. Also, the analysis of the peroxidase activity of rice Hb1 compared to that from horseradish peroxidase (HRP) showed that the catalytic efficiency of rice Hb1 for the oxidation of guaiacol using H _2_O _2_ as electron donor is several orders of magnitude lower than that of HRP ( *k*_cat_/ *K*_m_ = 15.8 and 44,833 mM ^-1^min ^-1^, respectively). Additionally, it was observed that recombinant rice Hb1 poorly protects *E*. *coli* from H _2_O _2_ stress ^124^. This evidence indicates that it is unlikely that rice Hbs function *in vivo* as peroxidases.

Based on gene expression ( Table 1), protein localization and structural and kinetic properties of rice Hbs and data from the analysis of other plant and non-plant Hbs it is likely that Hbs play a variety of roles in rice plants growing under normal and stressed conditions. These functions may include O _2_-transport, O _2_-sensing, NO-scavenging and redox-signaling. Future work on rice Hbs should focus on testing the above potential functions as well as newly proposed functions that emerge from novel observations.

## Evolution of rice hemoglobins

Hbs are widely distributed in land plants, ranging from primitive bryophytes to evolved angiosperms ^9^. The outline of plant Hb evolution subsequent to land colonization was clarified ^15^. Briefly, a phylogenetic analysis showed that plant and animal *hb* genes diverged 900–1400 mya, that land plant *nshb* and *thb* genes vertically evolved through different lineages from algal ancestors, that nsHbs-1 and nsHbs-2 are monophyletic and evolved via a gene duplication event prior to the divergence of monocots and dicots at ca. 140 mya, and that symbiotic *hb*s originated from *nshb* genes at ca. 94 mya. Likewise, the structural analysis of primitive nsHbs and Lbs revealed that changes during the evolution of nsHbs to Lbs were a hexacoordinate to pentacoordinate transition at the heme prosthetic group, a length decrease at the CD-loop and N- and C-terminal regions, and a compaction of the protein into a globular structure ^47, 125^.

In contrast, the evolution of rice Hbs is partially understood owing to the limited availability of Hb sequences from a wide variety of wild and cultivated rice. However, the outline of monocot Hb evolution is rather well understood. Thus, in this section we will discuss the evolution of rice Hbs within the context of major events that occurred during the evolution of monocot Hbs. A major event during the evolution of land plant nsHbs was the duplication of an ancestral *nshb* into *nshb-1* and *nshb-2* prior to the monocot-dicot divergence ^15, 126^. Sequence analysis revealed that *nshb-1* and *nshb-2* genes exist in dicots and that apparently only *nshb-1* genes exist in monocots ^9, 80, 127^. Earlier Garrocho-Villegas and co-workers ^62^ reported the existence of a nsHb (Hb5) divergent from rice (Hb1 to 4) nsHbs-1 and suggested that nsHbs divergent from nsHbs-1 evolved within monocots. Subsequent phylogenetic analysis of monocot nsHb sequences revealed that apparently only *nshb-1* evolved within monocots, that *nshb-1* duplicated early in the evolution of monocots originating clade I and clade II *nshb*s ( *nshb*s *-I* and *nshb*s *-II*, respectively), that nsHbs-I correspond to dicot nsHbs-1, and that nsHbs-II diversified into regular nsHbs-II, post-helix H-containing nsHbs-II and 11 amino acids deletion-containing nsHbs-II ^51^. This analysis also showed that *O*. *sativa* var. indica and *O*. *sativa* var. japonica Hb1 to 4 and Hb5 cluster within clade I and clade II, respectively, and that *O*. *glaberrima* and *O*. *rufipogon* (whose all *nshb* copies remain unidentified because their genome sequencing is in progress) nsHbs cluster within clade I. Thus, apparently clade I and clade II lineages remain conserved during the evolution of rice nsHbs ^51^.

Evaluation of the rate of divergence of selected land plant Hbs revealed that evolutionary rates slowed down previous to the origin of magnoliophyta and that the rate of divergence was slower in rice Hb1 than in rice tHb ^128^. This observation suggested that rice Hb1 (and conceivably other rice nsHbs) evolved under the effect of the stabilizing selection. However, the estimation of the variability of the *O*. *sativa* var. indica, *O*. *sativa* var. japonica, *O*. *glaberrima* and *O*. *rufipogon nshb* and *thb* genes revealed that in these plants variability is higher in *nshb*s than in *thb*s and that these genes evolved under the effect of neutral selection ^52^. Currently the effect of rates of divergence and gene variability on the Hbs function during the rice evolution is not known.

## Concluding remarks and future directions

In the preceding sections of this review we summarized major findings from the study of rice Hbs. This review also reveals some major lacunae in our ability to completely understand rice Hbs, more specifically the lack of information about the precise functions of Hbs in rice organs. The proposed functions for rice Hbs are mostly based on the analysis of other plant and non-plant Hbs. Thus, future work should evaluate the Hb activities ( *e.g*. the NO-binding and -detoxifying activities) in either rice organs or rice cell cultures under a variety of growing conditions. Elucidating the functions of rice Hbs also requires the identification of organic molecules and protein partners that interact with rice Hbs. Other lacunae are the absence of biochemical, biophysical and cellular data on the properties of rice Hb2 to 5 and tHb. Generating recombinant rice Hb2 to 5 and tHb should provide Hb polypeptides for a variety of analyses that reveal the biochemical and biophysical properties of these proteins.

With the exception of rice *hb2*, a lacuna is the absence of experimental information about the *cis*-elements and *trans*-acting factors that regulate the expression of rice *hb*s. This information may help to integrate the *hb* gene expression into the rice metabolisms, including those that are modulated by plant hormones.

A final lacuna is the incomplete understanding of the evolution of rice Hbs. Sequencing of the *O*. *glaberrima* and *O*. *rufipogon* genomes will be completed soon and most likely a number of rice genomes (including that of *O*. *barthii*, which is postulated as the ancestor of *O*. *glaberrima* ^129, 130^) will be sequenced within the near future. This will provide new Hb sequences for phylogenetic analysis and the understanding of the evolution of rice Hbs, including the identification of ancestral rice Hbs and the evaluation of the effect of rice domestication and breeding during the evolution of rice Hbs.
