# Supplementary material for: Rice ( Oryza) hemoglobins
Source: F1000Res. 2014 Dec 11;3:253. Originally published 2014 Oct 27. [Version 2] doi: 10.12688/f1000research.5530.2 (PMC4304225; doi:10.12688/f1000research.5530.2)
Supplement: Peer review history

# Peer review: Referee response for version 2

**DOI:** 10.5256/f1000research.6325.r7011

**Published:** 2014-12-19

**Authors:** Juliette T. J. Lecomte, Eric Johnson

**Affiliations:** 1 T. C. Jenkins Department of Biophysics, Johns Hopkins University, Baltimore, MD, USA

We appreciate the authors consideration of our comments and we agree with the revisions to this paper.  This review effectively highlights the current state of knowledge for this field and points out additional research that would make valuable contributions.

We have read this submission. We believe that we have an appropriate level of expertise to confirm that it is of an acceptable scientific standard.

# Peer review: Referee response for version 1

**DOI:** 10.5256/f1000research.5905.r6522

**Published:** 2014-11-20

**Authors:** Juliette T. J. Lecomte, Eric Johnson

**Affiliations:** 1 T. C. Jenkins Department of Biophysics, Johns Hopkins University, Baltimore, MD, USA

The manuscript “Rice (Oryza) hemoglobins” by Arredondo-Peter, Moran and Sarath reviews the current status of research on the group of proteins found within rice plants belonging to the hemoglobin (Hb) superfamily. There is growing interest in Hbs, particularly non-mammalian Hbs, owing to their potential role as mediators of reactive nitrogen molecules, such as nitric oxide. Such a review is welcome, as rice is an important cultivar and adaptation to stress during growth plays an important role in the survival of the plant. The review covers a wide range of relevant topics, such as phylogenetic aspects, cellular localization, and chemical properties.

Within this review, the literature on rice Hbs is covered thoroughly, as witnessed by the extensive bibliography that accompanies the manuscript.  However, closer inspection of that bibliography reveals an underlying issue with this manuscript. Although over 150 citations are given, a minority of those papers reference research performed specifically on rice plant or rice Hbs. This is not meant as a slight against the authors, rather as an observation that current research on rice Hbs is still in its infancy and extensive literature on the topic simply does not exist. With that in mind we would suggest altering this manuscript to acknowledge this shortage of experimental evidence in the following ways.

- By far the majority of experimental evidence exists of Hb1, yet throughout the manuscript Hb1 is treated more-or-less equally to Hb2-Hb5. The authors should focus on Hb1 in presenting the available evidence, then in a separate section (perhaps “correlations to other Hb genes”) the authors could examine homology models and hypothetical behavior without the danger of having conjecture misinterpreted by the reader as published fact.
- tHb belongs to a separate class of Hbs than the other Hbs in rice (Hb1 through Hb5), and throughout the manuscript it is often treated as an afterthought. As an addendum to the above comment we would also suggest a separate section be reserved for tHb. Though little experiment information may be available for this protein, a short stand-alone section would be more informative than a series of trailing paragraphs.
- Great care must be taken by the authors when postulating biophysical characteristics of rice Hbs using only homology models based upon a single crystal structure.  Simple variations in structure can carry significant changes in heme iron coordination, reactivity and binding affinity.  If such conjecture is used in this manuscript it must be plainly stated with the caveat that this is not published fact, or experimental evidence.

 

Additional specific comments

 

1. To orient the reader it would be helpful to include a two-panel figure containing (a) the amino acid sequence alignment of Hbs 1 through 5 using Hb1 as the anchor, and (b) the alignment of tHb using Arabidopsis thaliana GLB3 as the anchor. In both panels, the differences between each Hb and Hb1 (a) or tHb and GLB3 (b) could be emphasized (e.g., with color). The secondary structure of Hb1 (a) and GLB3 (b) could be indicated as well. In the text the % sequence identity for the relevant pairs should be mentioned. In addition, are the japonica and indica sequences identical? Whether one or the other cultivar is used should be clearly indicated throughout the review (for example, in Figure 2).
2. Apoprotein folding pathways are discussed on page 7. An additional statement as to the biological relevance of the folding pathway and information about association with the heme (when, where) would be interesting.
3. In general, it is not possible to predict the thermodynamic or chemical properties of a heme protein based on its primary structure. The case of *Parasponia andersonii* and *Trema tomentosa* hemoglobins provides one illustration of the difficulty within the plant world. Predictions can be inaccurate even when a three-dimensional structure is available. This shortcoming of sequence analyses and modeling could be emphasized with a discussion of specific examples and used to advocate the need for additional hemoglobin research.
4. Reaction with nitric oxide is a likely function of many hemoglobins. According to the work of Gardner and colleagues, the NO dioxygenase reaction begins with the binding of dioxygen followed by combination with NO to produce nitrate. In this mechanism, binding of NO to the iron is not necessary. In this regard, the statement on page 3 (bottom left) should be clarified.  Likewise, the Hb/NO cycle as proposed by Igamberdiev and Hill involves oxyHb. In contrast, the description on page 9 suggests that one turnover occurs with oxyHb, followed by formation of Hb-NO.
5. Speculations regarding the formation of homodimeric or heterodimeric structures should also be qualified, since the concentration of the hemoglobins (sub micromolar) appears to be much lower than the projected Kd (mM).
6. In the "Postulated functions” section, a proposal is made that Hb5 and tHb are O _2_ transporters.  Could the authors elaborate on this function? (Transport to what and for what purpose, and is it consistent with the cellular concentrations?)
7. Ligand binding is central to the function of hemoglobins. The “kinetic” section provides little such information while the second paragraph of the “Postulated functions” section offers numbers. It would be useful to consolidate the kinetic data with a table containing the measured equilibrium and rate constants for Hb1 and Hb2 (CO, NO, O2), as published in various primary references (for example, reference 97), and for Hb relatives mentioned in the text.

We have read this submission. We believe that we have an appropriate level of expertise to confirm that it is of an acceptable scientific standard, however we have significant reservations, as outlined above.

# Response

**Published:** 2014-11-29

**Authors:** Raul Arredondo-Peter

**Affiliations:** Universidad Autonoma del Estado de Morelos, Mexico

We thank Drs. Lecomte and Johnson for evaluating the first version of this review and for providing useful comments and suggestions. We agree with them and incorporated the suggested changes into the revised version of the review.

# Peer review: Referee response for version 1

**DOI:** 10.5256/f1000research.5905.r6520

**Published:** 2014-11-13

**Authors:** Martino Bolognesi

**Affiliations:** 1 Department of Biosciences, University of Milan, Milan, Italy

The Review by Arredondo-Peter *et al.* presents our current knowledge on the fascinating field of plant non-symbiotic (nsHb) and truncated hemoglobins (tHb), for which substantial, but scattered, information has accumulated over the past twenty years. The Review deals specifically with rice Hbs. Work from several distinct worldwide groups has so far provided information on the gene families for five nsHbs, and for a single tHb in rice; moreover, expressed proteins have been located to various plant organs and developmental stages. Crystal structures and kinetic analyses have helped delineating the potential roles of rice Hbs in plant physiology, highlighting different O _2_ binding affinities that differentiate the various Hbs. A main question that remains unanswered concerns the *in vivo* functions carried over by the five distinct rice nsHbs and tHb; hints reviewed from the literature include O _2_ sensing, signaling, O _2_ transport, NO scavenging, and NO dioxygenase pseudo-enzymatic activities (others may also be plausible). The Review includes evolutionary considerations (and the effects of rice selection through domestication) that will be reinforced by the forthcoming completion of rice genomes.

Overall, the Review provides a useful compendium over a subject whose reunification into a coherent presentation will indeed support deeper exploration of the functional aspects, whose scientific and practical relevance cannot be underestimated.

I have read this submission. I believe that I have an appropriate level of expertise to confirm that it is of an acceptable scientific standard.

# Response

**Published:** 2014-11-16

**Authors:** Raul Arredondo-Peter

**Affiliations:** Universidad Autonoma del Estado de Morelos, Mexico

We thank Dr. Bolognesi for evaluating this review and his comments.
